# Decreased Serum Brain-Derived Neurotrophic Factor Concentrations 72 Hours Following Marathon Running

**DOI:** 10.3389/fphys.2021.668454

**Published:** 2021-07-15

**Authors:** Astrid Roeh, Stefan Holdenrieder, Julia Schoenfeld, Jan Haeckert, Martin Halle, Peter Falkai, Johannes Scherr, Alkomiet Hasan

**Affiliations:** ^1^Department of Psychiatry and Psychotherapy, Klinikum der Universität München, Ludwig-Maximilians University Munich, Munich, Germany; ^2^Department of Psychiatry, Psychotherapy and Psychosomatics of the University Augsburg, Bezirkskrankenhaus Augsburg, Medical Faculty, University of Augsburg, Augsburg, Germany; ^3^Institute of Laboratory Medicine, German Heart Centre Munich, Technical University Munich, Munich, Germany; ^4^Department of Prevention and Sports Medicine, Klinikum Rechts der Isar, Technische Universitaet München, Munich, Germany; ^5^Deutsches Zentrum für Herz- und Kreislauf-Forschung (DZHK) e.V. (German Center for Cardiovascular Research), Partner Site Munich Heart Alliance, Munich, Germany; ^6^University Center for Preventive and Sports Medicine, Balgrist University Hospital, University of Zurich, Zurich, Switzerland

**Keywords:** exercise, cognition, biomarker, aerobic, running

## Abstract

**Background:** Physical exercise has been linked to beneficial effects on brain plasticity. One potential key mechanism for this relationship is an exercise-induced increase of brain-derived neurotrophic factor (BDNF). However, the kinetics of BDNF in athletes during training phase, extreme exercise competition, and recovery period have not been investigated so far.

**Methods:** We assessed serum BDNF concentrations in 51 marathon runners (23% female, mean age 43 years) in a longitudinal study design over a period of 6 months. Assessments were conducted during the training period before the marathon and after the marathon race during short-term (24 to 72 h) and long-term (3 months) follow-ups. Potential confounders (fitness level, sex, and platelet count) were included in subsequent linear-model analyses.

**Results:** Linear mixed-model analyses revealed a main effect of time for BDNF concentrations over the study period (*F*_(4,89.389)_ = 4.296, *p* = 0.003). Values decreased significantly with the lowest values at 72 h after the marathon compared to baseline (*p* = 0.025), a finding that was more pronounced in the larger male cohort.

**Conclusion:** Prolonged exercise induces a significant decrease in serum BDNF concentration 72 h post-exercise. We assume that this observation is mainly driven by regenerative mechanisms and a higher muscular utilization.

## Introduction

Numerous animal and human studies have demonstrated positive effects of exercise on various dimensions of brain plasticity (Roig et al., 2013; Northey et al., 2018; Stern et al., 2019). Different possible mechanisms seem to contribute to this finding, ranging from vascular adaptations and metabolic effects to changes in levels of neurotrophins and cytokines (Voss et al., 2013; Suzuki, 2019). Brain-derived neurotrophic factor (BDNF) is involved in neural plasticity mechanisms such as in an increased neuronal growth, improved synaptogenesis, and neuronal survival (Tolwani et al., 2002). The exercise-dependent release of BDNF has been discussed to be one of the key mechanisms linking the exercise effects and improved cognition in rodents and humans (Hamilton and Rhodes, 2015; Szuhany et al., 2015). Even though primarily central BDNF concentrations are assumed to influence cognitive performance, peripheral plasma and serum concentrations have also shown a positive correlation to central concentrations in rodents (Angelucci et al., 2011). In addition, human studies have indicated a close relationship between peripheral BDNF plasma and serum concentrations and central effects (e.g., reduced plasma and serum concentrations in patients with depressive disorders and bipolar disorders that returned to baseline after treatment; Polyakova et al., 2015). Moreover, BDNF does cross the blood–brain barrier (Pan et al., 1998), supporting the notion that peripheral BDNF concentrations can be considered as a proxy of central levels.

A meta-analysis (Dinoff et al., 2017) of 55 studies (eight studies with resistance training exercise and the other studies with aerobic training exercise, mean exercise duration of 38.9 ± 37.5 (7–240) min, 75.4% male; mean age of 27.9 ± 10.8 y; mean body mass index (BMI) of 24.0 ± 1.7 kg/m^2^) revealed an approximate 60% increase in BDNF concentrations after a single bout of acute exercise in both resistance and aerobic study designs. Higher effect sizes were seen during longer durations of exercise, for higher cardiorespiratory fitness (VO_2_max) and for male participants. The increase in the studies seemed to be transient, with decreased values back to baseline levels after 15–60 min post-exercise. So far, investigations of BDNF kinetics during longer follow-up phases after acute exercise are lacking.

Effects of exercise training programs (repeated bouts of exercise) on BDNF concentrations have widely been investigated. One recent meta-analysis (Marinus et al., 2019) with older participants (>60 years, 17 included studies, either aerobic or strength exercise or a combination of both) analyzed exercise programs of 8 to 24 weeks (16 studies) and single bouts of exercise (one study). Baseline values of BDNF were comparable between exercise and control groups. After both single aerobic or strength exercise as well as after strength or combined aerobic/strength exercise programs, BDNF concentrations increased significantly. Training periods for a marathon event were not explicitly studied in prior research, but might have similar effects as exercise training programs with both designs displaying repeated bouts of exercise.

As displayed in these two meta-analyses, many studies investigated acute effects of exercise on BDNF concentrations and the effects of exercise programs, but the subsequent exercise durations were moderate. Longer durations of exercise or excessive exercise have rarely been discussed. In one prior study (Rasmussen et al., 2009) with a similar exercise duration compared to marathon running, BDNF concentrations increased two- to threefold during 4 h of rowing (8 participants, last measurement 1 h after cessation of the exercise). In active elderly marathon runners and bicyclists (*N* = 56 athletes and 58 sedentary controls), BDNF concentrations were similar to the sedentary controls (not measured immediately after a marathon; Winker et al., 2010).

Although the majority of studies discussed BDNF as one possible key mechanism in explaining the positive effects of different forms of exercise on brain plasticity and cognition, many questions remain to be elucidated. Namely, no study to date has examined the kinetics of BDNF during a short- and long-term follow-up period after prolonged exercise. It is unclear whether the transient increases of BDNF after acute exercise remain stable during the follow-up period. If we accept the relationship between increased levels of BDNF and improved brain plasticity with beneficial effects on cognition and functioning, it is crucial to understand the overall BDNF kinetics after acute exercise. This especially applies to the kinetics after excessive and prolonged exercise. Therefore, the aim of this study was to investigate BDNF levels in marathon runners, both in the training period, after the marathon and sequentially during the recovery period.

## Materials and Methods

Our study cohort was part of the Running effects on Cognition and Plasticity (ReCaP) trial. The detailed study protocol was previously published (Roeh et al., 2020). In brief, the ReCaP trial was a longitudinal study with participants of the Munich Marathon in 2017. Inclusion criteria were age between 18 and 60 years. All participants had to have completed at least one half-marathon in the past, possess sufficient German language skills, and be able to provide written informed consent. Exclusion criteria were relevant neurological, cardiac, or psychiatric diseases, pregnancy, and cannabis abuse.

**Figure 1:**
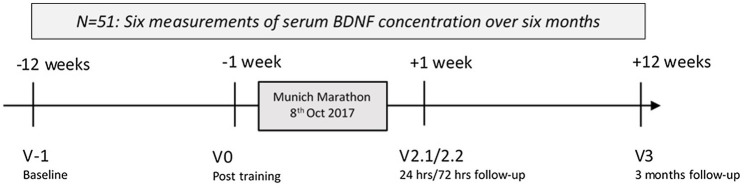
Study time: Longitudinal measurement of serum brain-derived neurotrophic factor (BDNF) concentrations around Munich Marathon 2017. Hrs, hours.

The study timeline is presented in Figure 1 and displays the six measurements over 6 months. Participants were assessed 12 weeks before (*Visit -1*), 1 week before the marathon (*Visit 0*), and shortly after successful completion of the marathon on 8th of October 2017 (*Visit 1*). At *Visit 1*, all measurements were performed within 2 h of the individual completing the marathon. The short-term follow-up contained measurements at 24 (*Visit 2.1*) and 72 (*Visit 2.2*) hours after completing the marathon, and the long-term follow-up was set at 12 weeks after the marathon (*Visit 3*).

Participants were asked to refrain from long-distance runs in the 3 days prior to the visits to increase the significance of the marathon event. The core hours for the visits were between 9:00 a.m. and 3:00 p.m., which also accounted for the visit immediately after the marathon event. All study participants underwent a standardized physical examination. BMI was calculated as kg/m^2^. Blood pressure (mmHg) was measured after resting in supine position for 5 min and before blood sampling. Blood samples were taken from the antecubital vein.

Total body fat percentage was measured by the skinfold caliper technique of Brozek (Brozek et al., 1963) at seven measure points and calculated with the formula of Brozek and Jackson (Jackson et al., 1980). Daily activity level of participants as an assumption for fitness levels was evaluated with the International Physical Activity Questionnaire (IPAQ; Craig et al., 2003).

All measures were obtained in the premises of the Department of Prevention and Sports Medicine of the Technical University Munich, except for the visit immediately after the marathon.

### Ethics and Registration

The study was performed in accordance with the guiding principles of the Declaration of Helsinki 2008, local laws, and regulations. The study protocol had been approved by the ethics committees of both the Ludwig-Maximilians University Munich (approval number 17-148) and the Technical University Munich (approval number 218/17 S). The study was registered at https://www.drks.de/ (DRKS-ID: DRKS00012496). All participants provided written informed consent before inclusion in the study.

#### Human Free BDNF Immunoassay

We measured serum and not plasma BDNF concentrations as serum measurements have been used more frequently than plasma measurements in previous studies, which improves comparability of our results (serum concentrations combine blood-borne bound and unbound BDNF, and plasma concentrations display only the unbound proportion; Dinoff et al., 2017). BDNF was measured in participants with at least four of six successful visits in serum by the human free BDNF ELISA kit (R&D Systems, Minneapolis, MN, United States) according to the instructions of the manufacturer with a dilution factor of 1:3 (standard and sample). All standards, samples, and controls were run once. A sigmoid curve was fitted on the resulting data, and the sample concentrations were calculated by the Dynex DS2 software.

Platelet counts were measured with Sysmex fluorescence flow cytometry (XE-2100D, Sysmex Xtra 2/2008) according to the instructions of the manufacturer and in line with the ICSH reference methods (International Council for Standardization in Hematology).

In a second step, results of serum BDNF concentrations and platelet count were corrected according to the method of Dill and Costill for dehydration (Dill and Costill, 1974; Alis et al., 2015).

### Statistical Analysis

Analyses were performed using SPSS 25 (IBM, Armonk, NY, United States) with a significance level of *α* = 0.05. Changes of BDNF concentrations and platelet counts for the within-subject factor “time” were evaluated by general linear mixed-models (LMM) with a diagonal covariance matrix in subjects with complete BDNF data for all visits. In case of a significant main effect of “time,” *post hoc* contrasts were calculated by testing a given measurement against baseline and adjusted for multiple comparisons (Sidak correction). We further conducted a sensitivity analysis by applying LMM for the time visits V0, V2.1, and V2.2 to display the short-term changes of BDNF between 24 and 72 h after the marathon. Moreover, we wanted to exclude the time point *visit 1* (within 2 h after the marathon), as we might have missed the acute changes after the marathon (As displayed, they peak at around 30 min after cessation of exercise.)

For evaluating possible moderators of BDNF (dependent variable) over the course of the study period, we thereafter included sex (between-subject factor) and physical activity levels (IPAQ scores, covariate) in a linear model analyses (repeated measures ANOVA) and adjusted for multiple comparisons (Sidak correction). Finally, for evaluating the influence of platelet count on BDNF concentration, we applied Pearson correlation for each visit and adjusted for multiple testing [Bonferroni correction with *α* = 0.008 for six visits (0.05/6)].

**Figure 2:**
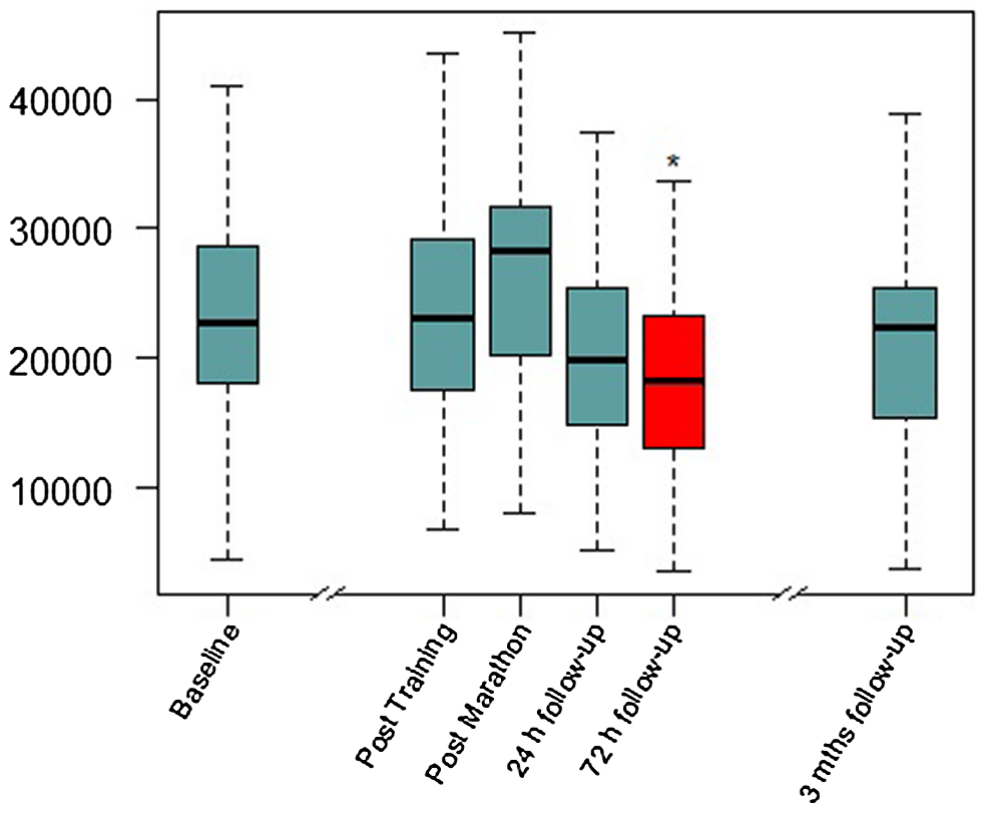
Serum BDNF concentrations over the study period. Mths, months. Red color and ^*^, significant difference compared to baseline visit in Linear mixed-model (LMM) analysis (details in Table 1).

Figure 2 was produced with R version 4.0.2 (R Foundation for Statistical Computing, Vienna, Austria).

**Table 1:** Serum BDNF concentration and platelet count over the study period; mean and SD.

	N	V-1 Baseline	N	V0 Pre	N	V1 Post	N	V2.1 24 h post	N	V2.2 72 h post	N	V3 3 months post	F	df	p	p V0/V-1	p V1/V-1	p V2.1/V-1	p V2.2/−1	p V3/V-1
BDNF (pg/ml)	51	23291.7 (8532.8)	51	23863.9 (8775.0)	51	26494.6 (8917.3)	51	19476.8 (7425.6)	51	18252.1 (7851.1)	51	20862.9 (8413.1)	6.692	5,98.076	<0.001	1.0	0.645	0.342	0.037	0.914
Platelet count (/μl)	51	222.1 (53.6)	51	226.3 (54.2)	51	265.0 (55.2)	51	220.1 (52.8)	50	218.3 (52.8)	51	231.7 (58.9)	5.234	5,87.319	<0.001	1.0	0.002	1.0	1.0	0.999

Effect of time and LMM analysis of each visit vs. baseline (V-1). Main effect of time (LMM) with F, df, p. In case of significant effect of time in the LMM, *post hoc* comparisons with Sidak correction are presented for each visit vs. baseline (V-1).

## Results

Of the initially included 100 participants, 71 successfully completed the marathon and 59 participants were examined at *visit 3* (last measurement at the end of the six-month study period). Complete data sets of six visits with BDNF levels were obtained in 51 participants [male/female = 42:9, mean age 42.7 ± 10.2 y, mean BMI = 23.4 ± 2.5 kg/m^2^, mean blood pressure systolic/diastolic at baseline (V-1) = 122.8/80.7 ± 11.3/6.4 mmHg, mean IPAQ at baseline (V-1) = 6299.8 ± 6040.7, mean training kilometers/week at V-1 = 42.3 ± 21.6, mean number of prior marathon events = 2.1 ± 5.1, mean marathon finishing time female/male = 264.7 ± 48.0/231.1 ± 31.4 min, and mean IPAQ at last visit (V3) = 5038.7 ± 5857.5]. At *visit 2.2*, results for blood count were missing in one participant due to a technical error, so at that time point, N for platelet count (and corrections for plasma volume shift) was 50.

Body fat percentage was 15.4 ± 4.8% in male and 22.2 ± 5.4% in female participants. Descriptive statistics and the complete test statistics are presented in Table 1 Corrected values for BDNF and platelet count (Dill and Costill method) are presented in Supplementary Table 1, and the results were not significantly changed.

Linear mixed-model revealed a significant effect of time (*F*_(4,98.389)_ = 4.206, *p* = 0.003), indicating BDNF concentration changes over the study period. In the short-term follow-up, the values decreased to levels below baseline with the lowest value at *visit 2.2* (72 h after the marathon, *p* = 0.025). At the last visit 3 months after the marathon event, there was no significant difference compared to baseline. Maximal values were reached at *visit 1* (not significant compared to baseline), shortly after the marathon. Figure 2 shows a visualization of BDNF (total) values over the whole study period. In a sensitivity analysis for short-term follow-up without the visits V-1, V1, and V3, the results of the minimal values 72 h after the marathon were confirmed (*p* = 0.003). Also, *visit 2.1* at 24 h after the marathon showed a significant decrease compared to *visit 0* (*p* = 0.036). See Table 2 for detailed results including platelet count (no significant changes over these visits).

**Table 2:** Sensitivity analysis: serum BDNF concentration and platelet count over the visits V0 to V2.2 (before the marathon and the short-term follow-up), excluding visits V-1 and V3; mean and SD.

	N	V0 Pre	N	V2.1 24 h post	N	V2.2 72 h post	F	df	p	p V0/V2.1	p V0/V2.2
BDNF (pg/ml)	51	23863.9 (8775.0)	51	19476.8 (7425.6)	51	18252.1 (7851.1)	6.078	2,103.117	0.003	0.036	0.003
Platelet count (/μl)	51	226.3 (54.2)	51	220.1 (52.8)	50	218.3 (52.8)	0.312	2,99.292	0.0732		

Effect of time and LMM analysis of each visit after the marathon vs. V0. Main effect of time (LMM) with F, df, p. In case of significant effect of time in the LMM, *post hoc* comparisons with Sidak correction are presented for each visit vs. V0.

### Exploratory Analysis of Influencing Factors

The LMM for the platelet count showed a significant effect of time: (*F*_(5,87.319)_ = 5.234, *p* < 0.001). Platelet count reached its maximum at *visit 3* shortly after the marathon (*p* < 0.001; see Table 1). We calculated Pearson correlations of serum BDNF concentration and platelet count (*N* = 51) for each visit to further examine a significant interaction. With the adjusted significance level after Bonferroni correction for multiple testing (*α* = 0.008), no correlation reached significance (V-1: *r* = 0.212, *p* = 0.135; V0: *r* = 0.240, *p* = 0.089; V1: *r* = 0.304, *p* = 0.030; V2.1: *r* = 0.094, *p* = 0.511; V2.2: *r* = 0.321, *p* = 0.023; V3: *r* = 0.380, *p* = 0.006). This points to different and not directly related kinetics of both factors.

As displayed, prior research has indicated higher effect sizes of BDNF increases after acute exercise in male participants and in participants with higher fitness levels. In the repeated measures ANOVA with dependent variable BDNF, the between-subject factor sex, and the covariate IPAQ score, analyses confirmed a significant effect of time with *F*_(5,235)_ = 4.704, *p* < 0.001. The interactions of BDNF × IPAQ (*F*_(5,235)_ = 1.437, *p* = 0.212), and of BDNF × sex (*F*_(5,235)_ = 1.142, *p* = 0.339) were not significant.

## Discussion

For the first time, we evaluated the kinetics of BDNF levels in healthy marathon runners in a longitudinal design over 6 months. The study period included the intensive training period prior to the marathon (3 months, *visit -1*), a visit in the tapering phase around 1 week before the marathon event (*visit 0*), measurements within 2 h after crossing the finishing line (*visit 1*), and short-term (24 h, *visit 2.1* and 72 h, *visit 2.2*) as well as long-term (3 months, *visit 3*) follow-up periods. BDNF concentrations decreased significantly below baseline levels at *visit 2.2* (72 h post-competition).

### Acute Effects Immediately After Cessation of Exercise

Prior literature has primarily focused on acute effects of exercise on BDNF levels. Evidence accumulated that BDNF significantly increases after acute exercise and that higher cardiorespiratory fitness could moderate this effect with higher increases in the group of higher cardiorespiratory fitness (Dinoff et al., 2017). The release of peripheral BDNF after acute exercise is mediated by various possible mechanisms. BDNF can be released by platelets (Fujimura et al., 2002), peripheral blood mononuclear cells (Brunelli et al., 2012), skeletal and cardiac muscle (Matthews et al., 2009), and endothelial cells (Prigent-Tessier et al., 2013). After cessation of exercise, peripheral BDNF concentrations rapidly (serum levels faster than plasma levels) return to baseline values (within 30–60 min; Yarrow et al., 2010; Gilder et al., 2014).

Due to our time frame for blood examinations within 2 h after crossing the finishing line, we might have missed the early peak of BDNF levels. Therefore, our results cannot directly be compared to prior findings. Even though we observed a BDNF increase after the marathon, this was not significant (trend level).

### Decrease of BDNF in the Follow-Up Period

For the first time, we evaluated serum BDNF kinetics including a short-term follow-up period of 24 to 72 h after marathon running and we could demonstrate a significant decline of BDNF both in the analysis including all visits and in the sensitivity analysis with the visits 0, 2.1 and 2.2.

As prior studies did not apply such study design, our findings of decreased BDNF levels after 72 h cannot directly be compared to the existing literature. Nevertheless, two other studies revealed decreased levels within 1 h post-exercise: in one interventional study (*N* = 8 sedentary participants and *N* = 8 active participants) with three different intensities of exercise tests, a decrease of BDNF after maximal intensity exercise was observed only in the physically active group [30–60 min post-exercise (Nofuji et al., 2012)]. In another small interventional study (*N* = 26 trained cyclists), decreased levels of BDNF were detected 10 min after the first set and 60 min after the third set of sprint interval training in the experimental group after two and 6 months (Hebisz et al., 2019). Authors of both studies hypothesized regenerative functions of BDNF to contribute to the finding of decreased BDNF levels in terms of increased utilization of BDNF for muscle regeneration. This idea was based on the observation that BDNF knock-out mice showed delayed muscle regeneration (Clow and Jasmin, 2010). We can extrapolate this finding on our results, as muscle regeneration after prolonged exercise will require more time and therefore a higher (total) utilization of BDNF. Another possible mechanism to explain the unexpected effect of decreased BDNF levels could be an enhanced utilization by an upregulation of TrkB (tyrosine protein kinase) receptor in the peripheral tissues in the context of exercise (Gómez-Pinilla et al., 2002; Nofuji et al., 2012).

These mechanisms provide potential explanations of our findings of decreased levels of BDNF 3 days after a marathon, but comparability is reduced because of the different time points of BDNF assessments across our and the reference studies. However, a marathon with a mean duration of three to 5 h of mainly aerobic exercise needs longer regeneration periods and therefore the decreased BDNF levels could still be explained in this context. Future studies may add more follow-up assessments in order to thoroughly evaluate the BDNF kinetics between 72 h and 3 months after prolonged exercise. Such approaches will help to detect the first time point of normalized BDNF concentrations after such strenuous interventions.

### Exploratory Analysis of Influencing Factors

As recommended (Walsh and Tschakovsky, 2018), we simultaneously evaluated platelet counts in our cohort (see Tables 1 and 2). This approach is based on the observation that BDNF is stored in large proportions in platelets and that this platelet-bound proportion was discussed to contribute to the exercise-associated increase in serum concentrations (Fujimura et al., 2002). The pattern of the two parameters was different, and platelet count reached its maximum immediately after the marathon but did not similarly decrease in the follow-up period. Correlations for each visit were not statistically significant after correction for multiple testing. This pattern indicates that the platelet count does not offer an explanation for the BDNF kinetics in our study pointing towards independent dynamics.

Furthermore, we included sex and an estimation of fitness levels (measured with IPAQ) to covary our analyses. None of the two moderator variables had a significant impact on BDNF levels over the study period. However, the reader should be aware that the female sample size was much smaller compared to the male cohort (9 vs. 42), reducing statistic validity. Prior investigations displayed higher effect sizes of increased BDNF concentrations in male participants after acute exercise (Dinoff et al., 2017). In our cohort, the female participants had overall higher levels of BDNF, but the changes throughout the study period did not reach significance. Even though these results have to be interpreted cautiously because of the small sample size in the female cohort, a possible explanation for the difference of BDNF kinetics throughout the study period could be the result of differences in body composition (higher body fat percentage with 22.2 ± 5.4% vs. 15.4 ± 4.8% and lower muscle mass in female participants). If we accept the described hypothesis of higher muscular utilization of BDNF during regeneration, male participants with higher muscle mass would consecutively require higher amounts of BDNF. Our study cohort represents common marathon participants with largely male runners (in our cohort 23% female runners). Future studies should further investigate this difference with a more balanced cohort of male/female participants.

The association of regular exercise and BDNF concentrations shows highly variable results (Walsh and Tschakovsky, 2018) with more studies pointing to a more pronounced increase of BDNF in participants with higher cardiorespiratory fitness (Dinoff et al., 2017). We did not find an association between BDNF kinetics and IPAQ scores in our cohort. One possible explanation could be the homogenous fitness levels of our participants, as they were all well-trained and had experience in endurance competitions (see inclusion criteria).

### Limitations

We were able to display changes in serum BDNF concentrations in a large cohort of healthy marathon runners. However, we were not able not compare the BDNF results to a control group with no physical exercise over the study period of 6 months. Furthermore, our study cohort was homogenous in terms of fitness levels and heterogeneous in terms of sex (with larger sample sizes for male participants). Thus, we might have missed influences of fitness levels and sex on BDNF levels after the marathon. Even though interpretation of sex differences is limited because of the different sample sizes, we aimed to address this point with regard to prior findings in terms of an explanatory approach. Future studies should investigate a more heterogeneous cohort and include more female participants to help generalize the results.

As displayed, we measured BDNF levels within 2 h post-exercise and might have missed the maximal increase immediately post-exercise (which was found to peak 30–60 min post-exercise in prior studies). To account for this, we further applied a sensitivity analysis for the short-term follow-up period excluding *visit 1*. BDNF evaluations in an experimental design (e.g., immediate measurement of BDNF concentrations after a marathon on a treadmill) would help validate our results.

Even though we have provided new insights into the kinetics of BDNF after acute exercise, we can only hypothesize about the possible causal mechanisms at this stage. We could not exclude the possibility that very strenuous exercise can even have negative effects on the central nervous system (with a subsequent reduction in BDNF levels).

## Conclusion

We measured the kinetics of BDNF during a training period, in response to a marathon event and during follow-up in in a longitudinal naturalistic six-month study. We were able to show a significant decrease of BDNF concentrations in the marathon recovery period. One possible mechanism to explain this observation might be related to a higher BDNF utilization for muscular regeneration. Prior studies primarily examined the acute kinetics within the first hours after cessation of exercise, so this decline could not be displayed. Thus, based on our findings, future studies should focus on kinetics of BDNF and underlying mechanisms over a longer period of time rather than focusing on single post-exercise measures.

## Data Availability Statement

The raw data supporting the conclusions of this article will be made available by the authors, without undue reservation.

## Ethics Statement

The studies involving human participants were reviewed and approved by the Ethics Committees of both the Ludwig-Maximilians University Munich (approval number 17-148) and the Technical University Munich (approval number 218/17 S). The patients/participants provided their written informed consent to participate in this study.

## Author Contributions

AR, JoS, AH, and JuS designed the study. AR, JuS, JH, and SH collected data. AR, JoS, AH, MH, and PF interpreted and analyzed data. AR wrote the first draft of the manuscript. All authors revised the manuscript critically and provided consent of the final version.

### Conflict of Interest

The authors declare that the research was conducted in the absence of any commercial or financial relationships that could be construed as a potential conflict of interest.
