# Supplementary material for: Decreased Serum Brain-Derived Neurotrophic Factor Concentrations 72 Hours Following Marathon Running
Source: Front Physiol. 2021 Jul 15;12:668454. doi: 10.3389/fphys.2021.668454 (PMC8320388; doi:10.3389/fphys.2021.668454)
Supplement: Supplementary file 1 [file Table_1.DOCX]

|  | **N** | **V-1** Baseline | **N** | **V0** Pre | **N** | **V1** Post | **N** | **V2.1** 24 hours post | **N** | **V2.2** 72 hours post | **N** | **V3** 3 months post | **F** | **df** | **p** | **p**  V0/V-1 | **p**  V1/V-1 | **p**  V2.1/V-1 | **p**  V2.2/-1 | **p**  V3/V-1 |
| --- | --- | --- | --- | --- | --- | --- | --- | --- | --- | --- | --- | --- | --- | --- | --- | --- | --- | --- | --- | --- |
| **BDNF_c**  **[pg/ml]** | 51 | 23291.7 (8532.8) | 51 | 23866.3 (8728.5) | 51 | 26270.6 (8827.4) | 51 | 19772.9 (7495.7) | 50 | 18316.1 (7954.3) | 51 | 20919.2 (8699.9) | 6.274 | 5,94.665 | <0.001 | 1.0 | 0.741 | 0.359 | 0.046 | 0.929 |
| **Platelet count_c**  **[/µl]** | 51 | 222.1 (53.6) | 51 | 226.3 (53.9) | 51 | 262.8 (54.5) | 51 | 220.2 (52.5) | 50 | 218.2 (51.7) | 51 | 232.3 (58.4) | 4.858 | 5,94.267 | 0.001 | 1.0 | 0.004 | 1.0 | 1.0 | 0.999 |

Suppl. Table 1 BDNF_c and Platelet count_c represent results of correction for dehydration according to the method of Dill and Costill. Descriptive analysis over the study period, mean and (SD). Effect of time, LMM analysis of each visit versus baseline (V-1).
Main effect of time (LMM) with F, df, p. In case of significant effect of time in the LMM, post hoc comparisons with Sidak correction are presented for each visit versus baseline (V-1).
